# Pigmentation Affects Elastic Fiber Patterning and Biomechanical Behavior of the Murine Aortic Valve

**DOI:** 10.3389/fcvm.2021.754560

**Published:** 2021-12-10

**Authors:** Sana Nasim, Popular Pandey, Rosemeire M. Kanashiro-Takeuchi, Jin He, Joshua D. Hutcheson, Lidia Kos

**Affiliations:** ^1^Department of Biomedical Engineering, Florida International University, Miami, FL, United States; ^2^Biomolecular Sciences Institute, Florida International University, Miami, FL, United States; ^3^Department of Physics, Florida International University, Miami, FL, United States; ^4^Department of Molecular and Cellular Pharmacology, Leonard M Miller School of Medicine, University of Miami, Miami, FL, United States; ^5^Department of Biological Sciences, Florida International University, Miami, FL, United States

**Keywords:** atomic force microscopy, echocardiography, pigment, extracellular matrix, elastin

## Abstract

The aortic valve (AoV) maintains unidirectional blood distribution from the left ventricle of the heart to the aorta for systemic circulation. The AoV leaflets rely on a precise extracellular matrix microarchitecture of collagen, elastin, and proteoglycans for appropriate biomechanical performance. We have previously demonstrated a relationship between the presence of pigment in the mouse AoV with elastic fiber patterning using multiphoton imaging. Here, we extended those findings using wholemount confocal microscopy revealing that elastic fibers were diminished in the AoV of hypopigmented mice (Kit^Wv^ and albino) and were disorganized in the AoV of K5-Edn3 transgenic hyperpigmented mice when compared to wild type C57BL/6J mice. We further used atomic force microscopy to measure stiffness differences in the wholemount AoV leaflets of mice with different levels of pigmentation. We show that AoV leaflets of K5-Edn3 had overall higher stiffness (4.42 ± 0.35 kPa) when compared to those from Kit^Wv^ (2.22 ± 0.21 kPa), albino (2.45 ± 0.16 kPa), and C57BL/6J (3.0 ± 0.16 kPa) mice. Despite the striking elastic fiber phenotype and noted stiffness differences, adult mutant mice were found to have no overt cardiac differences as measured by echocardiography. Our results indicate that pigmentation, but not melanocytes, is required for proper elastic fiber organization in the mouse AoV and dictates its biomechanical properties.

## Introduction

The aortic valve (AoV) functions to maintain unidirectional blood flow between the left ventricle and the aorta for the systemic distribution. The AoV undergoes constant pressure, stress, flexure, biaxial tension, and compression during a cardiac cycle, which requires the AoV to be durable for ~3 × 10^9^ cardiac cycles over the course of an average lifetime. The AoV is composed of highly structured cellular and extracellular matrix (ECM) components required to maintain appropriate biomechanics. The AoV ECM is mainly composed of collagen, elastin, and proteoglycans. In the AoV, radially aligned elastic fibers and circumferentially aligned collagen fibers dictate AoV function (1). Pathological alterations in alignment and composition compromise AoV biomechanics. In human AoV, elastic fibers on the left ventricular side of the leaflets confer elasticity, allowing extension when the valve opens and recoiling when the valve closes. The proteoglycans in the internal spongiosa layer absorb compressive forces and lubricate interactions between the highly structured elastic fibers and fibrous collagen of the aortic side, fibrosa layer. The fibrosa layer with rich collagen fibers provides tensile strength to the AoV leaflets. Lastly, elastic fibers maintain the tissue load at low strains, allowing the collagen fibers and the leaflet to stretch passively before bearing the load at maximum loading (2). The structure-function relationship of elastin and collagen is critical for proper valve function but underscoring the need for a full understanding of leaflet fiber interactions and their dynamic behavior.

Along with the highly specialized ECM, cells within the AoV leaflets sense and respond to mechanical stresses and strains. The cellular responses to mechanical stresses play an important role in valve development and remodeling (3). Two major cellular components are found in the AoV leaflets: valvular endothelial cells (VECs) and valvular interstitial cells (VICs). The AoV leaflets consist of an outer layer of VECs on both fibrosa and ventricular sides that interact with blood and associated hemodynamic forces. The inner layers of VICs are responsible for producing and maintaining the ECM. The VICs comprise a heterogeneous population of cells that include smooth muscle cells, fibroblasts, myofibroblasts, neurons and glia (4, 5). Besides these subpopulations of VICs, murine heart valves contain melanin producing cells, the melanocytes (6). The location of these melanocytes coincided with another ECM protein, Versican B, in the atrioventricular valve (AV) leaflets, suggesting that melanocytes may regulate or be regulated by ECM molecules (7). Moreover, pigmented regions within the AV valves are significantly stiffer than non-pigmented regions establishing an association between pigment and the biomechanical properties of the valve (7, 8). Using nanoindentation, the elastic modulus of AV leaflets was found to be higher in hyperpigmented leaflets (11.5GPa) as compared to hypopigmented (5.5GPa) and wild type (7.5GPa) leaflets (8).

We recently showed that the elastic fiber network of the mouse AoV is affected by pigmentation (4). We compared the pigmented AoV of C57BL/6J wild-type with the AoV of two other mouse models that show variations in pigmentation levels. The K5-Edn3 transgenic mouse expresses the cytokine endothelin 3 (Edn3) under the control of the keratin 5 promoter (K5) leading to hyperpigmentation in cutaneous and non-cutaneous locations such as the heart valves. The Kit^Wv^ mouse harbors a spontaneous mutation in the receptor tyrosine kinase Kit locus leading to the complete lack of pigmentation in cutaneous and non cutaneous locations. Excessive pigmentation in the leaflets of the K5-Edn3 hyperpigmented mice associated with disorganized elastic fibers while the leaflets of the Kit^Wv^ hypopigmented mice had less elastic fibers when compared to wild-type mie as demonstrated by two-photon imaging. Given the changes in elastin fiber patterning and alterations in the biomechanical properties observed in the AoV leaflets of K5-Edn3 and Kit^Wv^ mice, a more detailed investigation of the valve phenotypes can lead to a better understanding of the underlying causes of AoV disease including fibrosis and calcification (9). The goal of this study was to further demonstrate the correlation between pigmentation and AoV leaflet stiffness and structure. We extended the analysis of the relationship between pigmentation and ECM organization by employing wholemount confocal imaging and another hypopigmented model, albino mice, where melanocytes are present but melanogenesis is blocked by a mutation in the pigment rate limiting enzyme tyrosinase. We also show how varying pigmentation levels affect regional stiffness using atomic force microscopy (AFM) on wholemount freshly dissected leaflets. Finally, we performed echocardiography to identify potential cardiac physiological manifestations resulting from the elastin phenotypes observed in the pigmentation mutant mice.

## Materials and Methods

### Animals

All mice were housed in the Florida International University Animal Care Facility. The animal protocol for this study was approved by the Institutional Animal Care and Use Committee (IACUC 19-017). IACUC regulations were followed throughout the study. C57BL/6J wild type mice (WT for simplicity; Stock number 000664), B6(Cg)-Tyr^c−2J^/J mice (albino for simplicity; stock number: 000058), and C57BL/6J-Kit^W−v^/J mice (Kit^Wv^ for simplicity (only the homozygous spontaneous mutants were used); Stock number: 000049) were purchased from Jackson Laboratory (BarHabor, ME). The K5-tTA; TRE-Edn3-lacZ (K5-Edn3, for simplicity) transgenic mice were generated in our laboratory (10). Ten to twelve-week-old mice (strains: WT, K5-Edn3, Kit^Wv^, albino) (*N* = 5–6; 3F and 3M) were utilized for the mechanical and functional assessment of the AoV leaflets.

### Genotyping

Genomic DNA was isolated from tail biopsies of K5-Edn3 mice. Genotyping was performed using the primers: 5′ CCAGGTGGAGTCACAGGATT 3′, 5′ ACAGAGACTGTGGACCACCC 3′, for the recognition of the K5-tTA gene and, 5′ GGCCTGTGCACACTTCTGT 3′, 5′ TCCTTGTGAAACTGGAGCCT 3′ for the TRE-Edn3-LacZ transgene. Routine PCR conditions were used (45 cycles of 94°C for 30 s, 60°C for 60 s, and 72°C for 60 s) with an initial 3 min hold at 94°C. PCR product was then visualized in 1.5% agarose gel containing 0.5 μg/ml ethidium bromide (Thermo Fisher Scientific, Pittsburgh, PA). The K5-tTA produced a 244 bp band, whereas the TRE-Edn3-LacZ produced a 463 bp band. WT, Kit^Wv^, albino were identified phenotypically and did not require a genotype assessment.

### ECM Structural Staining and Quantification

#### ECM Staining

Alexa Fluor 633 (AF 633) hydrazide (Invitrogen, Waltham, MA, Cat# A30634) for elastic fiber staining was used as previously described (11, 12). 0.2 μM concentration of AF633 probe in PBS was used along with 10 μM CNA-488 probe for collagen fiber staining (CNA-488 was kindly gifted by Chris Reutelingsperger from Maastricht University, Netherlands) (13). The wholemount AoV tissue staining was carried out for 45 min at room temperature prior to 4% paraformaldehyde (PFA) fixation and pigment bleaching in 10% H_2_O_2_ overnight. Tissues were then placed on a glass side with the three leaflets of the AoV facing up and sealed with a coverslip. Z-stack images were taken using an Olympus Confocal BX61 microscope.

#### Staining Quantification

The obtained Z-stack images were utilized for 3D reconstruction and image processing. In brief, Z-project plugin in NIH ImageJ (14) was used to compile the z-stacks, and the ROI manager plugin was used to quantify the fluorescence signals from each stack after tracing a region around the leaflet.

#### Angle Quantification

A spectral based Cytospectre tool box was used to quantify the orientation of the elastin and collagen fiber angles from the z-stack images (15). For angle quantification, high magnification regional images were taken in all the four mouse models (Supplementary Figure 1). Collagen and elastic fiber alignment were separately measured in the green and red channel, respectively. All the collagen fibers were used as reference to elastic fibers, where the collagen fiber angles for each of the mouse model leaflet was set to zero and elastic fibers were rotated accordingly.

### Atomic Force Microscopy

#### Mouse Aortic Valve Isolation and Setup

Ten to twelve-week-old mice (strains: WT, K5-Edn3, Kit^Wv^, albino) (*N* = 4–5) were euthanized and the hearts were immediately washed in cold PBS. Within 5 min, AoV leaflets were isolated and placed on a coverslip in a 60 mm petri dish. Coverslips were glued with clear silicon waterproof sealant to the petri dish to avoid any movement of the coverslip. During the analyses, leaflets were kept in cold PBS, and all measurements were taken within 30–45 min of leaflet isolation. Following the AFM measurements, all samples were fixed in 4% PFA for 5–10 min (Supplementary Figure 2).

#### AFM Set Up

An XE-Bio system (Park systems, Santa Clara, CA, USA) in contact mode was used for all AFM measurements. Pre-calibrated gold-coated bead AFM probe (Novascan Technologies, Ames IA, USA) with a 1 μm diameter SiO_2_ microsphere were used for the indentation measurements. The spring constant of the tip was 0.1 N/m. Force curves measurements at the targeted region of AoV leaflet were performed with a probe approaching speed of 0.3 μm/s and peak force range 0.4–2.4nN. Each targeted point was measured three to five times to ensure the accuracy of the measurement.

#### Data Analysis

The sample's Young modulus of elasticity (Y_E_) was calculated using a Hertz model:


$$\begin{array}{l} F=\frac{4Y_{E}R^{0.5}}{3\left(1-v^{2}\right)}\delta^{1.5} \end{array}$$

Where, *v* is the Poisson's ratio, which was set to be 0.5, and R of 0.5 μm is the radius of the tip. Sample indenting force (F) and deformation (δ) were calculated as follows:


$$\begin{array}{l} F=k\left(d-d_{0}\right) \end{array}$$

$$\begin{array}{l} \delta =\left(z-z_{0}\right)-\left(d-d_{0}\right) \end{array}$$

A custom MATLAB script was used to fit the measured force curves to obtain the Y_E_ values for each region.

#### Regional Pigment Quantification From Resected Leaflets

For the quantification of pigment, all the wholemount leaflets were oriented in the same direction and four regions were labeled: tip, belly, base, and commissure. All images were converted into 8-bit and each region was identified based on the intensity of the darker and lighter regions associated with pigment using ImageJ. Percent regional pigment was normalized with total leaflet pigment.

### Echocardiography

#### Animal Preparation and Imaging Setup

Prior to image acquisition, mouse chest fur was removed by applying hair removal cream. Each animal was shaved immediately before the image acquisition and placed under 1–2% isoflurane. For consistency and comparability, all imaging conditions were controlled between animals. Mouse body temperature was monitored and maintained around 37°C during the entirety of the protocol. Since cardiac function is influenced by heart rate (HR), the HR was maintained at a similar level (450 ± 50 bpm) within each strain during imaging. Additionally, echo measurements were performed at similar times after anesthesia was administered to minimize differences in anesthesia-related effects to cardiac function.

The imaging setup included applying warm gel on the shaved chest and placing the mouse on a heating platform. ECG was continuously monitored. The heart was imaged with MX400, 20–46MHz linear transducer, with an axial resolution of 50 μm. Images were taken at 300–400 frames per second. All the imaging were done using a Vevo 3,100 (FUJIFILM Visual Sonics, Toronto Canada) imaging system under the IACUC rules and regulation at Sylvester Comprehensive Cancer Center at University of Miami.

#### Echocardiographic Assessments and Data Analysis

M-mode images in the left ventricle short-axis view and B-mode long-axis view were obtained for each mouse in a blinded fashion. Pulsed wave Doppler images were taken by placing the sample volume parallel to flow direction, which was assisted by Color Doppler-mode during long-axis view into left ventricular outflow tract and ascending aorta for AoV function. For mitral valve assessment, the apical four-chamber view was used (Supplementary Table 1). Quantified measures of function were obtained by averaging three consecutive heartbeats during the echocardiographic examination. From the M-mode, the left ventricular anterior, posterior, and inner wall during systolic and diastolic peaks were taken to assess the LV thickness. Aortic valve measurements included peak AoV pressure, mean AoV velocity, peak AoV velocity, velocity across the AoV. Mitral valve Doppler was used to assess diastolic function by measuring intraventricular relaxation (IVRT)/contraction time (IVCT), the mitral peak velocity of early filling ratio to peak velocity flow in late diastole (E/A), and ejection time (ET). All data were analyzed using the Vevo Lab software (FUJIFILM Visual Sonics, Toronto Canada).

### Tail Cuff Measurements

A non-invasive small animal blood pressure monitoring system (CODA Kent Scientific, Torrington, CT) was used to measure heart rate, systolic and diastolic blood pressure, and mean arterial blood pressure in ten- to twelve-week-old mice. In brief, the CODA tail-cuff system uses a specialized volume-pressure recording sensor to determine the tail blood volume to measure the blood pressure. All animals were kept anesthetized under 1–2% isoflurane. 10–15 consecutive readings were taken per animal to obtain stable blood pressure measurements.

### Statistical Analysis

All quantitative data are given as mean ± standard error mean (SEM). For AFM results, each color point within the strain represents a biological replicate calculated from a mean of *N* = 3–4. “*N*” indicates the number of biological replicates within each strain and “*n*” indicates the technical replicates. For AFM and echocardiography results, statistical packages in GraphPad were used to assess data normality and variance between the groups (GraphPad Prism6 Software, LaJolla, CA). Statistical differences were determined using either the student's *t*-test or one-way analysis of variance (ANOVA) along with Tukey's *post hoc* HSD test with significance considered as *p* < 0.05. All the shown percent differences were normalized by the specified comparison.

## Results

### Elastic Fiber Patterning of The Aortic Valve Leaflets Is Affected by Pigmentation

**Figure 1:**
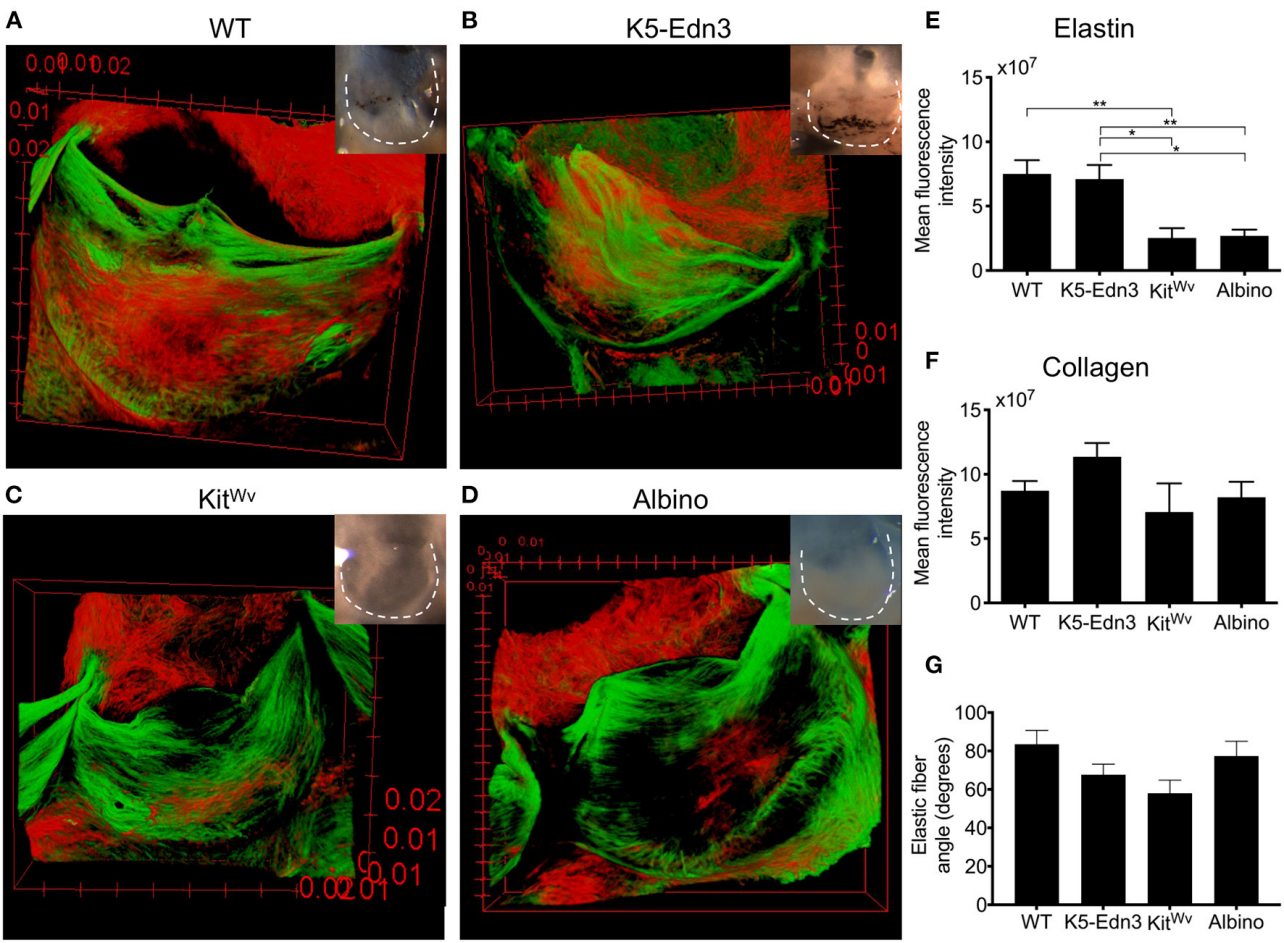
Relationship between pigment variation and ECM in the AoV leaflet. Elastin (red) and collagen (green) staining of 12–13 weeks old mouse wholemount AoV leaflets of **(A)** WT, **(B)** K5-Edn3, **(C)** Kit^Wv^, and **(D)** albino. Leaflets in the optical microscope images correspond to those in the images of the stained leaflets. Quantification of **(E)** elastin and **(F)** collagen fluorescence staining. **(G)** Elastic fiber angle quantification. MAG = 10X. Mean ± SEM shown; **p < 0.001, *p < 0.05. *N* = 4–5 biological replicates.

To assess the organization of elastic and collagen fibers in wholemount AoV leaflets, we used AF633 and CNA488, respectively. As in human AoV, murine wild type AoV had radially aligned elastic fibers and circumferentially aligned collagen fibers (Figure 1A). In the K5-Edn3 transgenic mouse Edn3 overexpression is under the control of keratin 5 promoter, resulting in excess pigment production by both cutaneous and non-cutaneous melanocytes (Figure 1B). The patterning of elastic fibers in the leaflets of K5-Edn3 mice appeared disoriented with fibers misaligned and ectopically located (Figure 1B). Kit^Wv^ and albino hypopigmented mice were assessed to further understand the role of pigment in elastic fiber patterning. Kit^Wv^ mice lack melanocytes in hair follicles leading to a white coat color, whereas albino mice have melanocytes that are unable to produce pigment due to a mutation in the *Tyrosinase* gene. The AoVs of both mutants are hypopigmented (Figures 1C,D). Compared to WT, Kit^Wv^ and albino AoV leaflets had fewer elastic fibers (Figures 1C,D). Quantification of elastic fiber fluorescence indicated that the elastic fibers was significantly reduced in the leaflets of Kit^Wv^ (66.3 ± 27.8%, *N* = 7, *n* = 3) and albino (64 ± 54.8%, *N* = 3, *n* = 8) compared to WT (*p* = 0.0026, *N* = 4, *n* = 9). In comparison to the leaflets of K5-Edn3 (*p* < 0.01, *N* = 4, *n* = 7), Kit^Wv^ (64.4 ± 29.8%) and albino (62 ± 56%) leaflets also had significantly reduced amounts of elastic fiber fluoresence (Figure 1E). We did not find statistically significant differences among the four groups for the collagen fiber staining (Figure 1F). Alignment of elastic fibers in relationship to the collagen fibers was also found to be mostly orthogonal in all four mouse models (Figure 1G). The elastic fiber alignment in WT was found to be 83.57 ± 7.18°, K5-Edn3 to be 67.74 ± 5.46°, Kit^Wv^ to be 58.08 ± 6.79° and lastly, albino to be 77.39 ± 7.72° with the collagen fibers referenced as 0°.

### Micromechanical Properties of the Aortic Valve Leaflets Are Affected by Pigmentation

**Figure 2:**
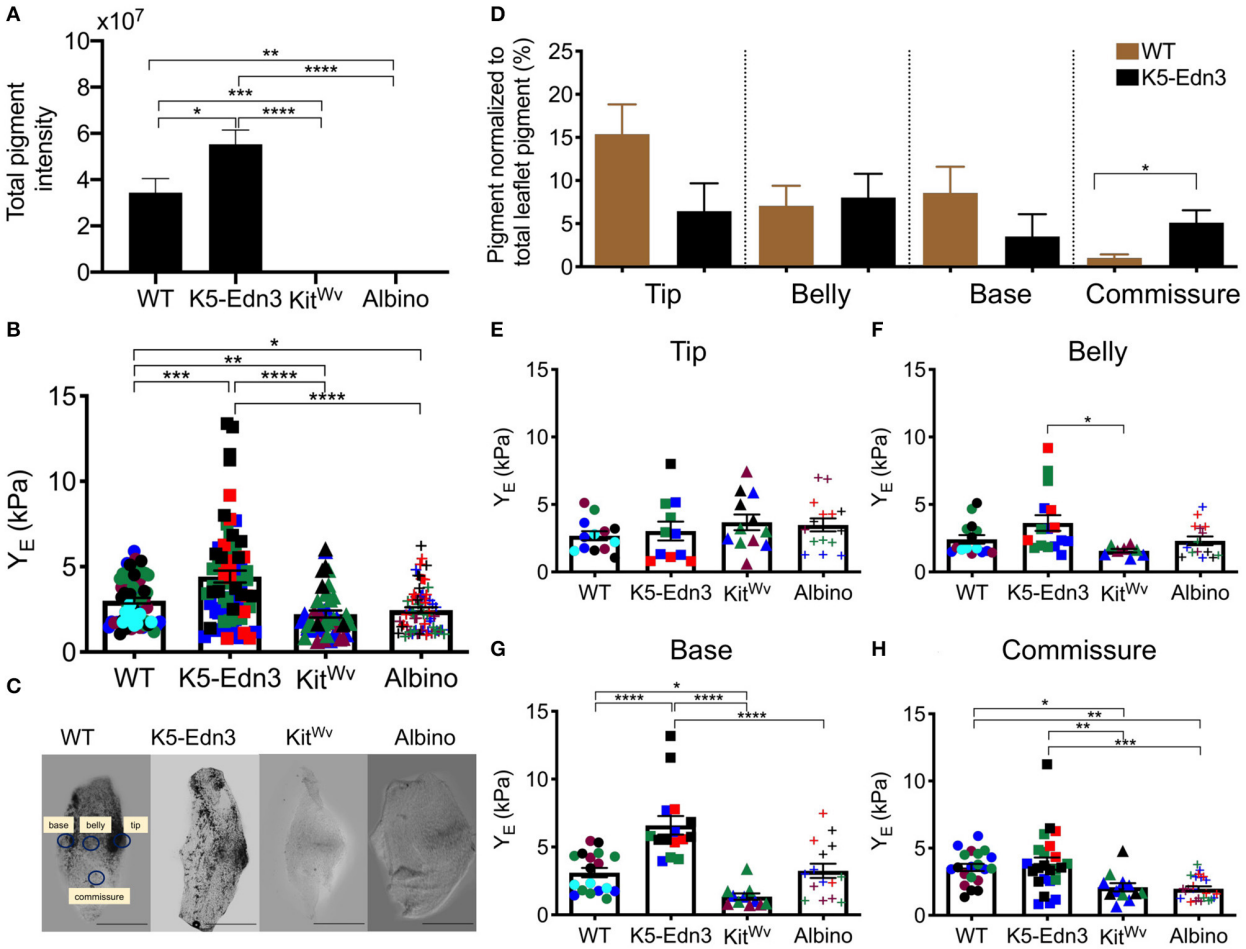
Relationship between pigment variation and stiffness of the AoV leaflet. **(A)** Total pigment in the wholemount AoV leaflets in the four mouse models. **(B)** Stiffness measurements of wholemount AoV leaflets of WT, K5-Edn3 (hyperpigmented), Kit^Wv^ and albino (hypopigmented) mice. **(C)** Optical microscope image of resected wholemount AoV leaflet from the four mouse models. All the four leaflets are oriented in the same direction. WT label of the four regions corresponds to the other three leaflets, and stiffness and pigment quantification **(D)** Regional pigment quantification in all four mouse models. Regional stiffness of wholemount AoV leaflets of the four moue models in the **(E)** Tip **(F)** Belly **(G)** Base **(H)** Commissure. Mean ± SEM shown; ****p < 0.0001, ***p < 0.0005, **p < 0.001, *p < 0.05; *N* = 4–5 biological replicates per condition. Each color represents a biological replicate, and each color point represents an average of technical replicates of *N* = 3–4. Scale bar = 100 μm.

As expected, we found K5-Edn3 to have significantly higher overall leaflet pigmentation as compared to WT (*p* = 0.023), Kit^Wv^ (*p* < 0.0001) and albino (*p* < 0.0001) (Figure 2A). Similar to our previously published observations in the AV valve (8) (7), the overall stiffness of the AoV leaflet of the hyperpigmented K5-Edn3 showed a 30.9 ± 34.6% (*p* = 0.0004) higher stiffness compared to that of WT. Whereas hypopigmented Kit^Wv^ and albino were found to have significantly lower overall stiffness of 28.6 ± 25.4% (*p* = 0.004) and 22.8 ± 28.3% (*p* = 0.0183), respectively, in the AoV leaflet as compared to WT (Figure 2B). Moreover, compared to K5-Edn3, the hypopigmented Kit^Wv^ (*p* < 0.0001) and albino (*p* < 0.0001) AoV leaflets were found to have significantly lower stiffness of 45 ± 44.6% and 41.06 ± 46.80%, respectively (Figure 2B).

To further explore the relationship between pigment and the associated stiffness in wholemount AoV leaflets, the regional distribution of pigment (Figure 2C) was quantified. In WT leaflets pigment was found to be significantly lower in the commissure region compared to the same region in K5-Edn3 leaflets (*p* = 0.0026). In the tip region, pigment there was more pigment in WT leaflets when compared to those of K5-Edn3 (*p* = 0.0837) (Figure 2D). Of note, pigmentation was distributed more uniformly throughout the K5-Edn3 leaflets.

Among the four mouse models, there were no significant differences in stiffness within the tip region (Figure 2E), whereas K5-Edn3 showed significantly higher stiffness in the belly region compared to Kit^Wv^ mice (*p* = 0.0205) (Figure 2F). In the base region, K5-Edn3 exhibited higher stiffness (*p* < 0.0001) compared to WT, Kit^Wv^, and albino mice (Figure 2G). Furthermore, in the commissure region, WT stiffness was significantly higher than Kit^Wv^ (*p* = 0.0432) and albino (*p* = 0.0047) mice. Lastly, regional commissure stiffness of K5-Edn3 mice was comparable to that of WT and significantly higher compared to that of Kit^Wv^ (*p* = 0.0075) and albino (*p* = 0.0003) mice (Figure 2H). Overall we did not find any differences among the biological replicates in the mouse models (Supplementary Figure 3).

### No Overt Aortic Valve Functional Differences Found With the Variation of Pigmentation

**Figure 3:**
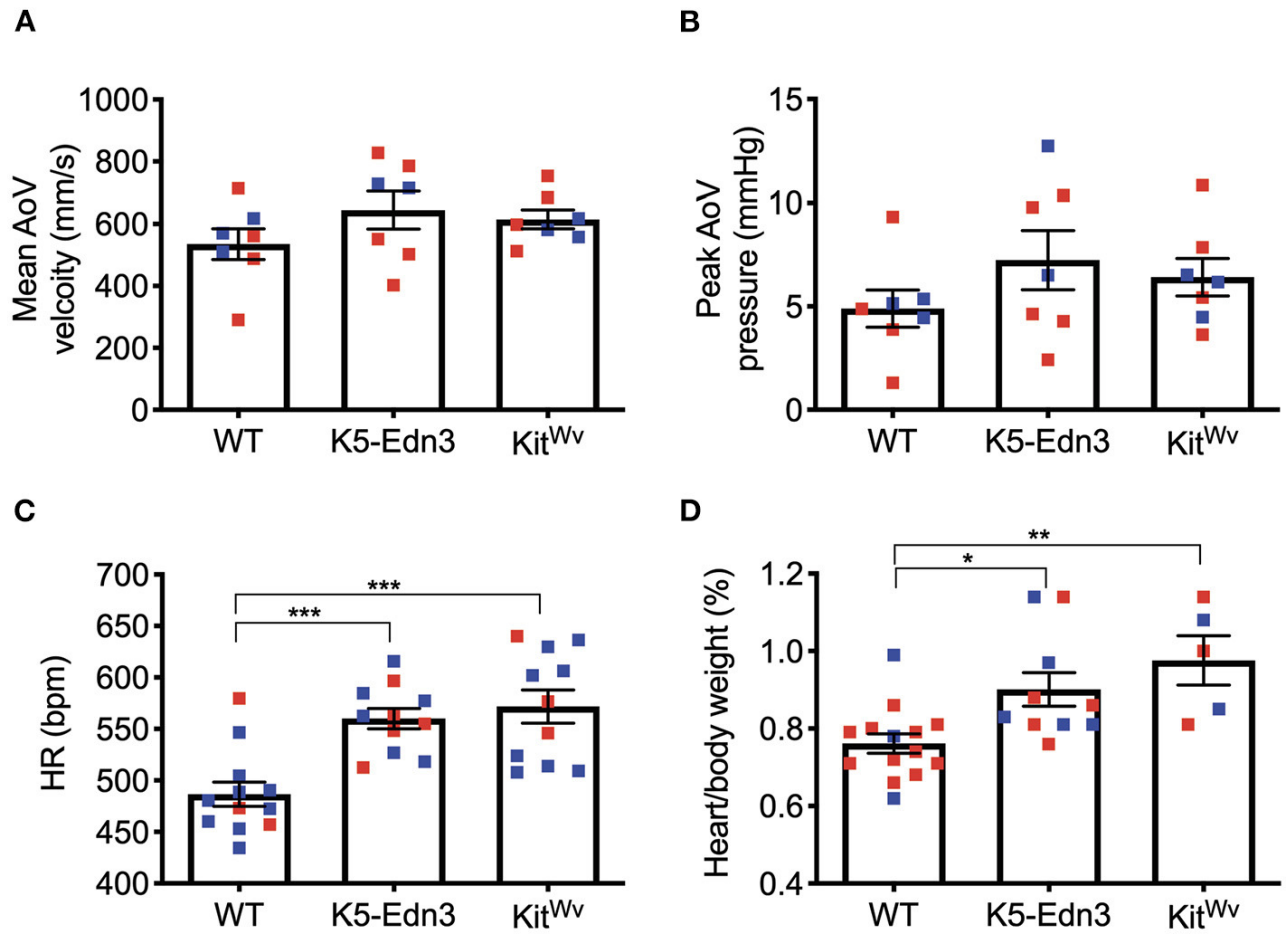
Functional cardiac assessment of mouse models with varying levels of pigmentation in the AoV (WT, K5-Edn3, Kit^Wv^). AoV blood flow measurement by pulse wave Doppler imaging **(A)** Mean AoV velocity **(B)** Peak AoV pressure **(C)** Non-invasive tail-cuff method to assess the heart rate. **(D)** Cardiac hypertrophy was determined by the ratio of heart weight (mg) to body weight (g). Mean ± SEM shown; ***p < 0.0005, **p < 0.001, *p < 0.05; *N* = 7 animals per group; Male (blue) and female (red); Age: 12–13 weeks old mice.

Using M-mode, B-mode, and pulsed wave Doppler imaging, cardiac functional parameters were assessed. No parameters traditionally associated with AoV function were found to be different between the mouse genotypes studied (Figures 3A,B). However, mitral valve ejection time was found to be significantly lower in K5-Edn3 mice compared to WT (*p* = 0.018) and Kit^Wv^ (*p* = 0.0126). There was also a significant difference (*p* < 0.05) between the E/A ratio of WT and Kit^Wv^ mice (Supplementary Table 2). We also assessed heart rate, systolic/diastolic blood pressure, and mean arterial pressures with conventional non-invasive tail-cuff blood pressure measurements. There was a significant increase in heart rate (*p* = 0.0001) and body weight/heart weight ratio (*p* < 0.01) among K5-Edn3 and Kit^Wv^ mice compared to WT mice (Figures 3C,D).

## Discussion

The AoV cells and ECM microstructure confer the appropriate biomechanical properties for valvular function. In the current study, we assessed wholemount AoV ECM patterning, specifically elastin and collagen in four different mouse models (WT, K5-Edn3, Kit^Wv^ and albino). We further show the biomechanical properties in the mouse models using AFM in freshly dissected wholemount AoV leaflets. The mechanical properties of murine AoV have been previously explored with other techniques such as micropipette aspiration and atomic force microscopy on fixed tissue sections (16, 17). This is the first study to report the relationship of ECM patterning and its associated biomechanical properties in freshly dissected wholemount AoV leaflets.

Using multiphoton imaging, we recently showed that the hypopigmented Kit^Wv^ mice lacked elastic fibers and had reduced elastin gene expression, whereas hyperpigmented K5-Edn3 mice with overabundant pigment had increased elastic fibers and elastin gene expression (4). The analysis presented here corroborates and extends these findings to another hypopigmented model, albino mice. We show that hypopigmented AoV leaflets (Kit^Wv^ and albino) have diminished elastic fibers. The addition of albino mice indicate that pigment production—rather than the presence of melanocytic populations—lead to the observed changes in elastic fiber patterning. We also found that elastic fibers in the WT leaflets were orthogonal to the collagen fibers, similar to the alignment known in human AoV (4, 18). While not significantly different, we observed trends that indicate less orthogonality in the K5-Edn3 and Kit^Wv^ elastic fibers. To be noted, although pigment has not been observed in human valves, cells expressing melanocyte phenotypic markers [including dopachrome tautomerase (DCT) and tyrosinase related protein 1 (TRP1)] have been observed (4). These melanocytic markers exist within leaflets in a manner that mirrors the localization observed in murine leaflets. Future studies are needed to fully understand how these cells contribute to AoV patterning across species. Understanding the mechanisms associated with elastogenesis is critical as elastin abnormalities result in congenital AoV defects and elastin degradation can initiate AoV disease (19–21). The emilin1 deficient mouse has been proposed as a model for human fibrotic aortic valve disease (9). Emilin 1 is an elastin-binding glycoprotein involved in the process of elastogenesis. Its deficiency is associated with early postnatal elastic fiber fragmentation in the AoV and leaflet stiffening prior to full onset of fibrotic disease (22). The elastic fiber phenotype of the mouse models used in this study and its association to the presence of pigmentation may offer another avenue for the identification of factors important in the progression of aortic valve disease and provide potential therapeutic targets.

Previously pigment has been shown to be important in conferring biomechanical properties of the porcine retinal pigment epithelium using AFM and spectroscopy (23). Melanin granules were shown to be critical in maintaining the stiffness of the epithelium layer which is important in the mechanical separation between the retina from the choroid. Using nanoindentation by AFM we showed that pigmented regions of AV leaflets are stiffer than non-pigmented regions, suggesting that pigment affects valve biomechanics (8). Moreover, we previously showed that hyperpigmented AV leaflets are stiffer than WT or hypopigmented valve leaflets, suggesting a contribution of pigment to the overall mechanical properties of murine heart valves (7). Here, we cooroborate the previous finding that hypopigmented leaflets are less stiff than WT and hyperpigmented leaflets by adding the albino hypopigmented model. These results further demonstrate that the presence of pigmentation affects the overall biomechanical properties, mainly the base and the commissure regions of the AoV leaflet. It has also previously been reported that the commissures of the human AoV have higher elastic modulus using uniaxial tensile testing (24). We found regional differences in stiffness among the mouse models suggesting that the changes in the biomechanical properties and ECM patterning could alter gross leaflet properties. We observed a strong positive correlation between total amount of pigment and leaflet stiffness in WT mice (Supplementary Figure 4A, *R*^2^ = 0.9582). The overall stiffness was higher in the hyperpigmented mice and lower in the hypopigmented mice, as expected (Supplementary Figure 4B, *R*^2^ = 0.5765). Individual differences in relation between pigment and stiffness, however, may indicate that the microstructural differences observed in these mice affect the stiffness beyond differences in pigmentation. AoV leaflets are heterogenous, which presents challenges for the localization of the AFM probe for consistent AFM measurements. AFM quantifies stiffness with micron spatial resolution; therefore, small differences in the chosen regions of interest could yield different results. Despite challenges with assessing small regions of interest for micromechanical testing, this is the first study to compare localized stiffness with corresponding ECM fiber patterning in freshly isolated wholemount murine AoV. Although we previously reported that unequal levels of pigmentation may occur amongst the left, right and coronary leaflets of the AoV (25), in this study all WT and K5-Edn3 leaflets that were used for biomechanical analyses were pigmented and we did not tract their anatomical position within the valve. We recognize this may have introduced some biases in the results and suggest that future studies take into consideration leaflet position to account for potential ontogenetic and histological differences among the three leaflets. Overall, this study has found a novel role for pigment in proper ECM deposition and biomechanics of the AoV.

Cardiac function assessed by echocardiography was found to be similar between the mouse models despite the drastic elastic fiber phenotypic difference observed. Cardiac imaging in mouse models are challenging due to their small size and high heart rate. We speculate that the lack of overt differences could potentially be explained by some compensatory mechanism that allows cardiac function to remain normal such as hypertrophy represented by an increase in HW/BW ratio (Figure 3D) or stiffer heart muscle, which we did not explore in this study. Alternatively, it is possible that differences between the groups would manifest in later adulthood or in response to pathological cues. In addition, in our study, we used males and females which could explain the lack of differences between mouse strains since sex differences have been reported in preclinical (26) and clinical studies (27, 28). A diet variation may be used to create pathological conditions; for example, mice could be put under different types of diets such as celecoxib to manipulate glucocorticoid signaling to create a more permissive environment for the appearance of cardiac diseases (29). To further access if there were differences related to early stages of valve diseases particularly VIC activation, we found that the tip region of Kit^Wv^ was mostly devoid of α-SMA positive cells, which were also few in the belly and base regions (Supplementary Figure 5). However, no significant differences in the number of α-SMA positive cells were detected among the three mouse models. Future studies should assess cellular-level differences at developmental timepoints that correspond with elastin synthesis to understand associated mechanisms. A recent study showed that the expression of ECM components such as decorin, osteopontin, Cthrc1 and Ddr1 which are involved in collagen metabolism as well as TGFb signaling change in an age dependent manner potentially underlying cardiac functional differences observed in older mice (30). Metalloproteinase driven elastic fiber degradation has previously been shown to contribute to heart valve mineralization leading to progression of calcific aortic stenosis (31). Future studies that explore the mechanisms associated with elastic fiber differences and implications in pathological valve remodeling could lead to new insight into AoV homeostasis and disease.

## Data Availability Statement

The raw data supporting the conclusions of this article will be made available by the authors, without undue reservation.

## Ethics Statement

The animal study was reviewed and approved by IACUC Florida International University.

## Author Contributions

SN designed the experiments, drafted the manuscript, conducted the aortic valve dissections, staining, confocal imaging, and AFM data analysis. PP conducted the AFM force curve measurements. RK-T imaged echocardiography and analyzed the echo data. JH supervised PP and provided feedback on AFM experiments. LK and JH equally designed the experiments, supervised SN and reviewed all the versions of the manuscript. All authors read and approved the final manuscript.

## Funding

This work was supported by the Florida Heart Research Foundation. SN was partially funded by Florida International University Graduate School. PP was partially supported by NSF CBET 1454544 and FIU CASE distinguished postdoctoralfellowship.

## Conflict of Interest

The authors declare that the research was conducted in the absence of any commercial or financial relationships that could be construed as a potential conflict of interest.

## Publisher's Note

All claims expressed in this article are solely those of the authors and do not necessarily represent those of their affiliated organizations, or those of the publisher, the editors and the reviewers. Any product that may be evaluated in this article, or claim that may be made by its manufacturer, is not guaranteed or endorsed by the publisher.
