# Supplementary material for: Pigmentation Affects Elastic Fiber Patterning and Biomechanical Behavior of the Murine Aortic Valve
Source: Front Cardiovasc Med. 2021 Dec 10;8:754560. doi: 10.3389/fcvm.2021.754560 (PMC8702816; doi:10.3389/fcvm.2021.754560)
Supplement: Supplementary file 1 [file Data_Sheet_1.docx]

**MATERIALS AND METHODS**

**Wholemount AoV immunofluorescence and quantification:**

AoVs (N=3) were obtained and fixed in 4% paraformaldehyde (PFA) for 30-45 minutes at room temperature and washed in 1X PBS twice. Tissues were then pigment bleached using 10% H_2_O_2_ overnight in 1X PBS. All the light microscope images were taken before the pigment bleach. Tissues were then washed and processed for wholemount antibody staining. In brief, tissues were permeabilized in 1% Triton X-100 in PBS for 45 minutes, blocked (10% goat serum, 0.3% Triton X-100, 1%BSA) for 2 hours at room temperature on a shaker followed by primary antibody (rabbit polyclonal to anti-alpha smooth muscle actin, 1:100, Abcam, Cat#ab5694) incubation at 4°C overnight on a shaker. The primary antibody was diluted in a dilution buffer containing 1% goat serum, 0.3%TritonX-100, and 1% bovine serum albumin (BSA). Tissues were thoroughly washed for 2 hours with 1X PBS at room temperature, before secondary antibody staining (goat anti-rabbit IgG, Alexa Fluor 594; 1:200; Invitrogen; Cat#A11012) for 1.5 hours. Lastly, tissues were washed for 2 hours with 1X PBS at room temperature, incubated in DAPI for 1-2 minutes, and mounted with a mounting medium. The AoV cusps were positioned such that the valve leaflets were faced upward as z-stack images were obtained using an upright Olympus Confocal BX61 microscope. All z-stacks were 3D reconstructed, and images were quantified using NIH ImageJ. For quantification, a threshold was applied to all the images to remove background noise. Watershed tool in ImageJ was used to separate individual nuclei and the ‘Analyze particle’ toolbox was used to quantify selected either DAPI or αSMA positive cells. All the data were normalized to all the DAPI positive counts in the respective region.

|  | **Image view** | **Image Depth** | **Image Width** | **Frame Rate** | **Gate Depth** | **Gate Size** |
| --- | --- | --- | --- | --- | --- | --- |
| B-mode | Longitudinal-axis (LAX) view | 12-15 mm | 9-12 mm | 200-250 | - | - |
| M-mode | Parasternal Short-axis (SAX) view at the level of papillary muscles | 10-15 mm | 6-8 mm | 250-350 | 8 -14 mm | 6-8 mm |
|  | | | | | | |
| PW Doppler-mode |  | **Sample Volume Depth** | **Gate Size** | **Doppler Angle** |  |  |
| Aorta | - | 8-11 mm | 0.85 mm | 45-60^○^ | - | - |
| Mitral | Apical four-chamber view | 7-11 mm | 0.85 mm | 3-16^○^ | - | - |

Supplementary Table 1. Cardiac imaging settings for B-mode, M-mode, and PW Doppler-mode measurements

| Cardiac functional parameters | | | |
| --- | --- | --- | --- |
|  | WT | K5-Edn3 | Kit^Wv^ |
| Fraction Shortening (%) | 30.97±9.32 | 34.78±12.06 | 28.51±8.26 |
| Stroke Volume (µL) | 31.99±8.21 | 29.22±6.14 | 31.18±5.19 |
| Ejection Fraction (%) | 59.97±10.59 | 64.31±8.80 | 57.79±8.11 |
| E/A ratio | 1.48±0.53 | 1.35±0.43 | 1.95±0.41 ^+^ |
| Ejection Time (ms) | 54.18±4.00 ^*^ | 42.27±9.15 | 54.85±7.21 ^#^ |
| Myocardium performance index | 0.62±0.06 | 0.88±0.57 | 0.56±0.08 |
| Left ventricular mass (mg) | 70.54±19.93 | 61.78±14.39 | 65.03±11.38 |
| Peak AoV pressure (mmHg) | 4.89±2.37 | 7.23±3.77 | 6.41±2.38 |
| Mean AoV velocity (mm/s) | 535.27±131.25 | 644.27±159.54 | 614.74±80.71 |
| VTI (mm) | 41.1±9.80 | 42.70±9.62 | 48.02±6.92 |
| Peak AoV velocity (mm/s) | 1094.13±288.26 | 1324.80±373.41 | 1265.75±227.69 |
| Velocity across the AoV (mm/s) | 1075.1±282.39 | 1302.37±363.41 | 1248.45±228.6 |
| Isovolumic contraction time (ms) | 15.3±2.8 | 15.42±4.3 | 13.36±1.94 |
| Isovolumic relaxation time (ms) | 18.64±2.64 | 18.13±8.26 | 17.12±2.4 |
| Left ventricular relative wall index | 0.33±0.05 | 0.38±0.1 | 0.34±0.06 |
| Left ventricular volume during diastole (µL) | 66.11±20.7 | 57.08±15.74 | 63.7±15.45 |
| Left ventricular volume during systole (µL) | 29.75±16.38 | 22.50±11.84 | 30.06±13.3 |
| Left ventricular internal diameter in diastole (mm) | 3.86±0.53 | 3.64±0.43 | 3.82±0.39 |
| Left ventricular internal diameter in systole (mm) | 2.7±0.69 | 2.41±0.64 | 2.75±0.55 |
| Left ventricular posterior wall thickness in diastole (mm) | 0.64±0.09 | 0.67±0.13 | 0.65±0.07 |
| Left ventricular posterior wall thickness in systole (mm) | 0.99±.0.18 | 1.07±0.19 | 0.94±0.22 |
| Interventricular septum in diastole (mm) | 0.68±0.11 | 0.62±0.08 | 0.63±0.07 |
| Interventricular septum in systole (mm) | 1.01±0.21 | 1.06±0.23 | 0.97±0.15 |
|  |  |  |  |
| Tail cuff measurements | | | |
|  | WT | K5-Edn3 | Kit^Wv^ |
| Heart rate (beats/min) | 486.57±40.94 | 560.05±32.64 ^***^ | 571.78±53.49 ^+++^ |
| Systolic pressure (mmHg) | 129.98±27.68 | 135.45±26.78 | 143.14±17.11 |
| Diastolic pressure (mmHg) | 109.99±28.72 | 109.52±25.50 | 120.67±13.60 |
| Heart weight to body weight ratio | 0.76±0.09 | 0.90±0.13 ^*^ | 0.97±0.14 ^++^ |

Supplementary Table 2. Functional assessment of mice with a variation of pigment in the aortic valve. ^*^compare WT vs. K5-Edn3 ^#^compare K5-Edn3 vs. Kit^Wv +^compare WT vs. Kit^Wv^. Mean ± SEM shown. N=7 animals per group.
