# Supplementary figures and images for: Pigmentation Affects Elastic Fiber Patterning and Biomechanical Behavior of the Murine Aortic Valve
Source: Front Cardiovasc Med. 2021 Dec 10;8:754560. doi: 10.3389/fcvm.2021.754560 (PMC8702816; doi:10.3389/fcvm.2021.754560)

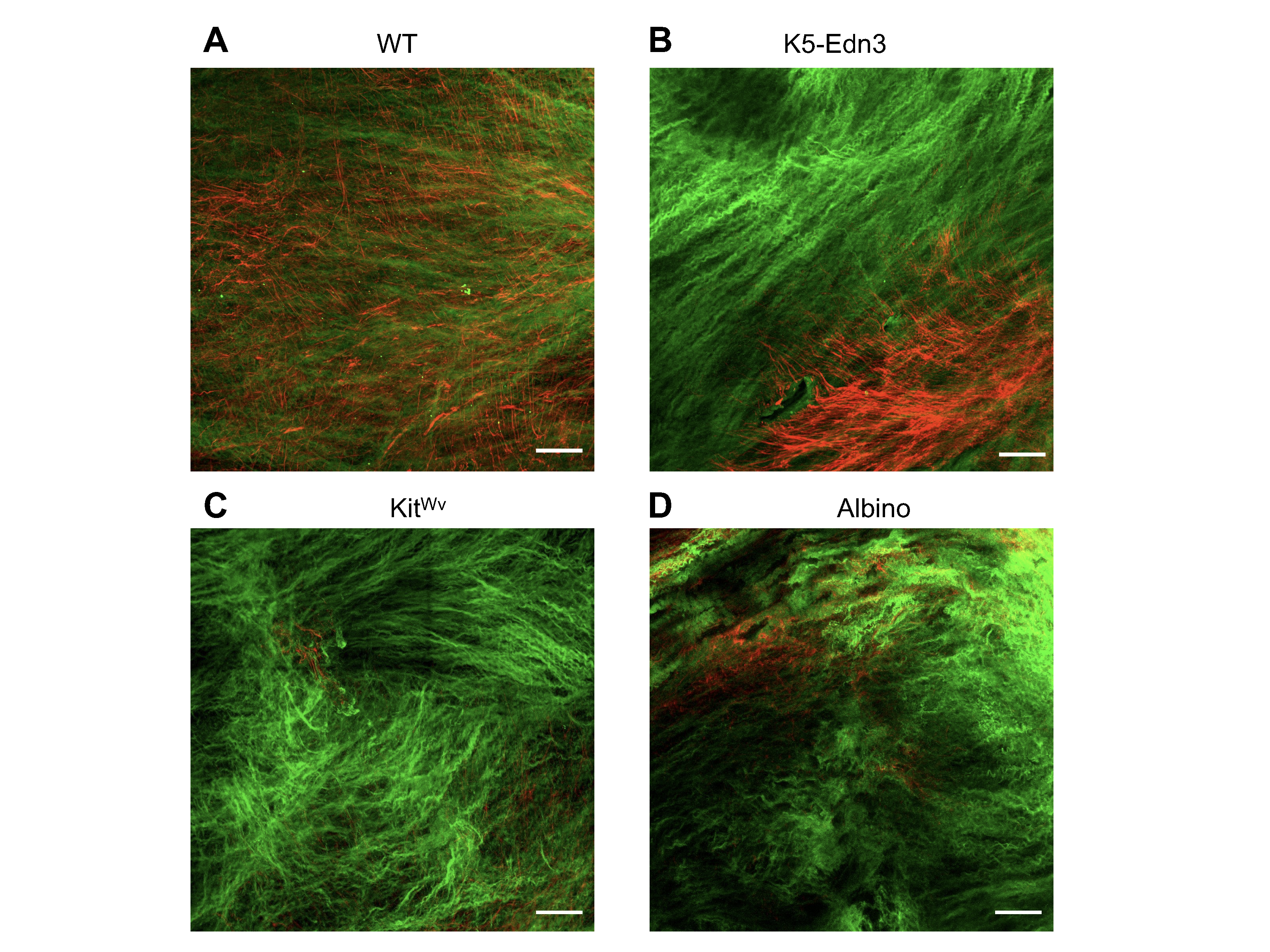

Supplement: Supplementary file 2 [file Image_1.TIFF]

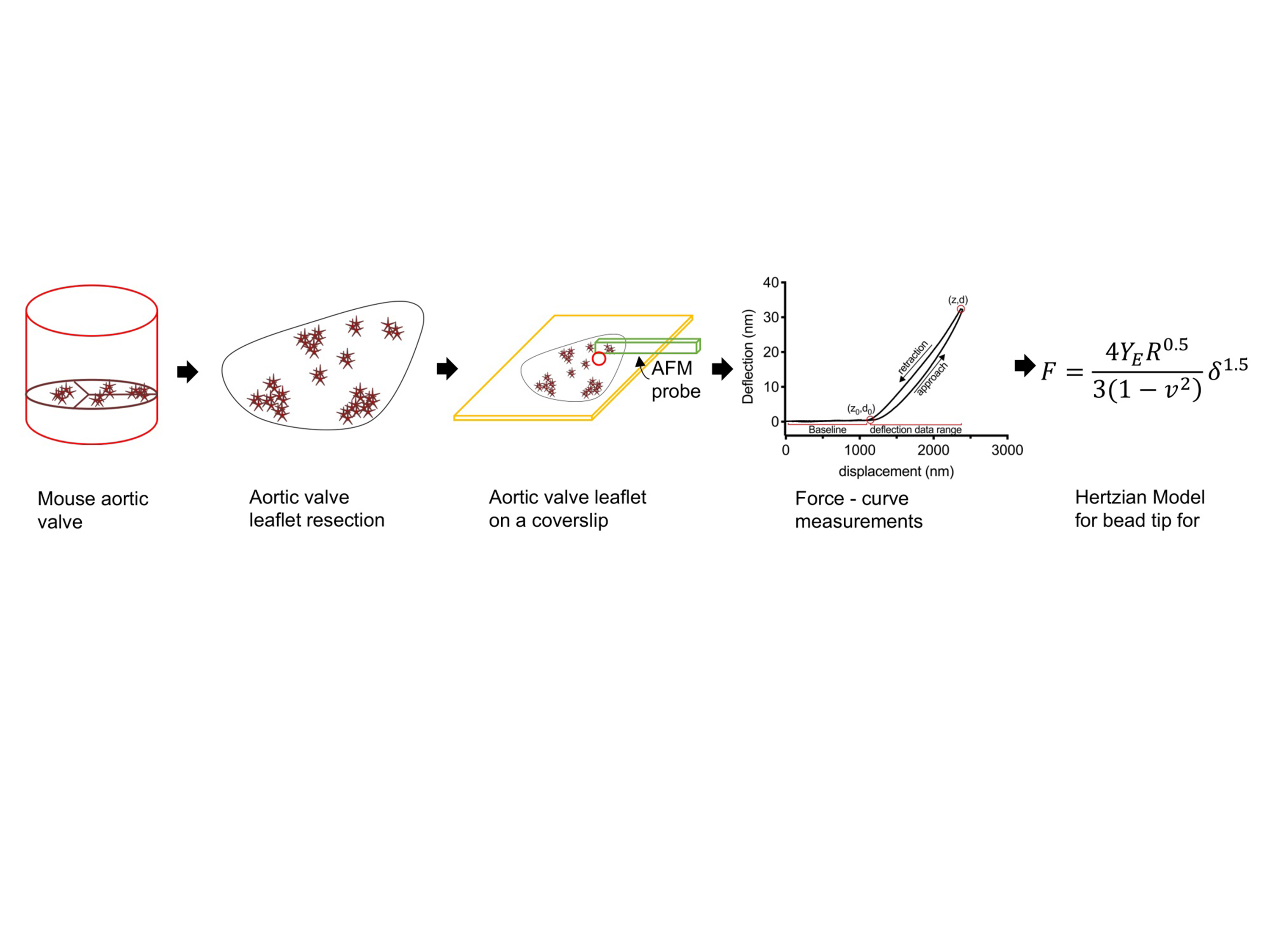

Supplement: Supplementary file 3 [file Image_2.jpg]

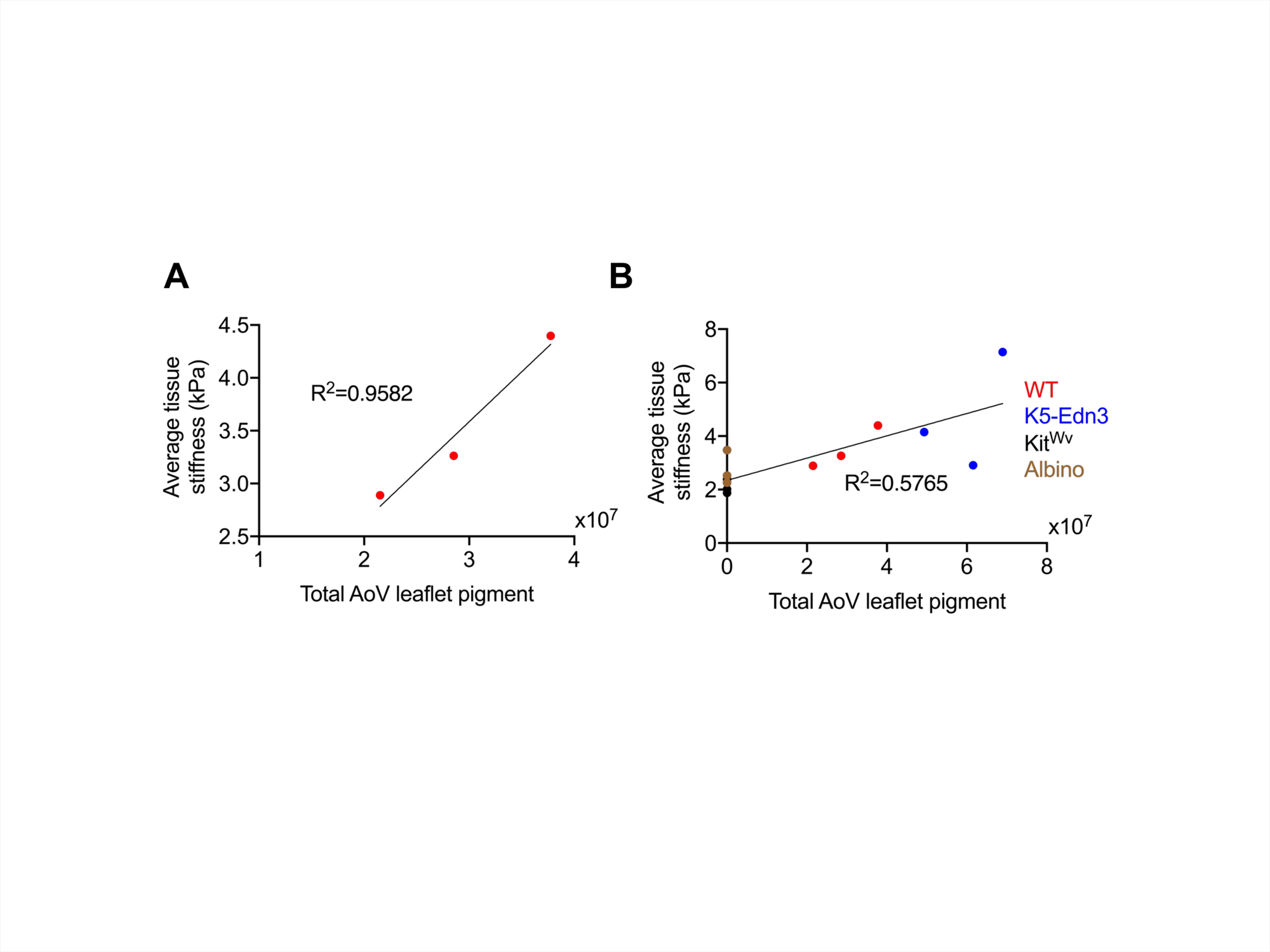

Supplement: Supplementary file 5 [file Image_4.TIFF]

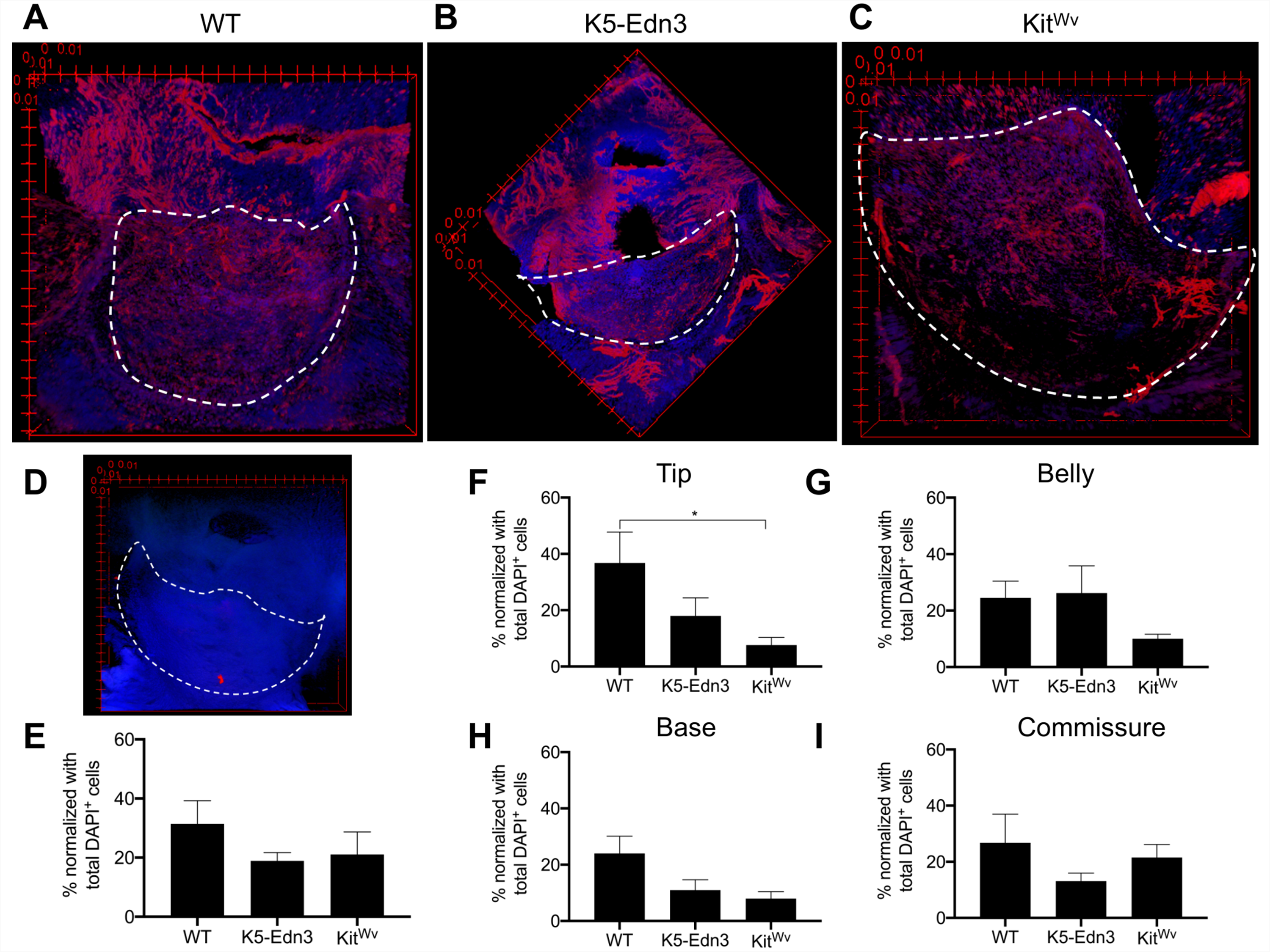

Supplement: Supplementary file 6 [file Image_5.TIFF]
